# Savings behaviour and livelihoods before and after COVID-19 – a four round panel dataset from Pune, India

**DOI:** 10.1038/s41597-026-06648-y

**Published:** 2026-03-02

**Authors:** Nitya Mittal, Sebastian Vollmer

**Affiliations:** 1 Centre for Modern Indian Studies, University of Goettingen, Göttingen, Germany; 2 Department of Economics and Centre for Modern Indian Studies, University of Goettingen, Göttingen, Germany

**Keywords:** Decision making, Economics

## Abstract

The data collected for this study focuses on two research question. First, it examines the effectiveness of a portable saving device in reducing temptation spending and increasing savings using a Randomised Control Trial (RCT) design. We then build on the data collected for RCT among slum dwellers in Pune, India and expand the scope of data collection to examine the long-term effect of the COVID-19 pandemic on livelihoods and consumption expenditure. Detailed information on income, savings, expenditure, knowledge about and behaviour during the pandemic was collected during various rounds. Additional information on female empowerment, decision making within the household and behavioural parameters was also collected. Four rounds of data were collected - two rounds before COVID-19 in 2018 and 2019 through field interviews, and two rounds in 2020 and 2022 through phone interviews. The baseline sample consisted of 1525 slum dwellers who earned above subsistence level income in Pune, and we have a balanced panel of 411 individuals.

## Background & Summary

Household savings are critical for welfare of low-income households^1,2^. However, due to several institutional, social and behavioural factors, the demand for savings among low-income households remains unmet^2,3^. This study examines the effectiveness of a saving promotion intervention through a randomised control trial (ID AEARCTR-0003682). The intervention aims to reduce temptation spending behaviour by providing a soft commitment product, a zip purse (Please see ref. ^1^ for more details).

A field experiment was conducted among slum-dwellers in Pune, India. A sample of 1525 individuals was interviewed at baseline (round 1) in 2018. Half of the respondents were randomly assigned to the treatment and received the portable saving device as well as a stationary lockbox. The remaining half formed the control group, and were provided with only a stationary lockbox. A post-intervention survey (round 2) was conducted in 2019 to evaluate the effect of the intervention.

A third round of data was collected through a follow-up survey in October-December 2020 (round 3) to examine the long-term impact of the intervention. As contact and mobility restrictions were imposed because of the COVID-19 pandemic, data was collected through phone interviews instead of field surveys, thus restricting the sample to those who had a phone and choose to receive the phone call. This round of data collection also allowed us to examine if the households who received the portable device were able to better cope with the shock due to the pandemic. We also extended the scope of the round 3 survey to examine the effect of the pandemic on livelihoods among the slum dwellers in Pune.

Many studies that evaluated the immediate socio-economic impact of the COVID-19 pandemic^4–15^ found that the lockdowns implemented to prevent spread of COVID-19 caused substantial economic distress, especially among vulnerable groups. However, since the adverse effects of shocks tend to persist over long periods, this study is among the few that examine how persistent the effect of the shock was, after the restrictions were lifted^16–23^. An additional round of data was collected in 2022 (round 4) via phone interviews to assess the socio-economic impact of the pandemic two years after its start.

This four-round panel dataset, spanning over 5 years, provides unique insights into the lives of individuals with just above subsistence level income in a developing country context^24^. The dataset can be used to understand the socio-economic behaviour of the sample individuals and examine how it changes temporally. Information on risk and time preference, and gender attitudes of individuals can provide a more nuanced understanding of such behaviours, especially when facing an unexpected shock. This is among the few datasets that could be used to study long-term causal impacts of the pandemic^24^. The dataset also provides insights about knowledge and behaviour of the individuals during the pandemic.

## Methods

The data was collected in four rounds. Round 1 data was collected in December 2018-January 2019, round 2 in August-October 2019, round 3 and round 4 data was collected through phone surveys in October-December 2020 and February-March 2022, respectively. The study was approved prior to field work by the Institutional Ethics committee at the University of Goettingen for all four rounds of data collection. Additionally, ethics approval was also obtained from the Institutional Ethics Committee at Indian Institute of Technology, Gandhinagar, India for the last two rounds of data collection. Participation in the survey was voluntary and informed consent was taken before start of each interview. Written informed consent was taken in round 1 and 2, while verbal (recorded) informed consent was taken in round 3 and 4 as the data was collected through phone interviews. The trial and a pre-analysis plan were registered in the American Economic Association’s registry for randomised controlled trials (ID AEARCTR-0003682).

### Sampling design

The sample was selected from two cities in Pune district (in Maharashtra, India), Pune and Pimpri-Chinchwad. Eight (out of 14) administrative wards from Pune and 6 (out of 8) administrative wards from Pimpri-Chinchwad were selected to draw the sample. More than 50 (254 settlements) and 80 (56 settlements) percent of total slum settlements in Pune and Pimpri-Chinchwad are located in these selected wards. The final sample was selected from about 32 slum settlements in Pune and about 10 slum settlements from Pimpri-Chinchwad. Most of the selected slum settlements in Pune were on private land, while most of those in Pimpri-Chinchwad were on land owned by state government. Most of these settlements were informal, and about a quarter of them lacked access to water and toilet facilities inside the house. Due to the nature of the trial, the study sample was selected from among those who earned more than their subsistence needs and therefore had the potential to save. Thus, individuals who indicated that they had at least some form of monthly income, which could come from permanent or informal work, remittances, or social welfare payments, were considered eligible for the study. Our sample thus represents poor households but not necessarily the ‘poorest of the poor’.

Enumerators conducted door-to-door visits in each of the selected settlements and recruited households that matched the above-mentioned criterion. To select study participants, we employed a random walk procedure within each slum. The enumerators started from a central landmark in each settlement, where they split in different directions and visited every second household. No monetary incentive was given to the participants for participation in the survey. We interviewed one person per household but the treatment was delivered to both spouses (see ref. ^1^). In round 1 we interviewed 1,525 male (18%) and female (82%) slum dwellers who were (i) 18 years and older, and (ii) had some income (either in the form of salary, remittances or social welfare transfers) at least once per week or on a monthly basis. The same individuals were re-interviewed in subsequent rounds.

### Survey instrument round 1

The survey instrument was organised in 10 sections in round 1. Section 1 focused on demographic and economic information about the respondent such as age, gender, marital status, education and, employment and income sources. It also included detailed questions about transfers received from and made to other people. In section 2 information regarding the demographic profile and assets owned by the household was collected. Household food consumption expenditure for a 30-day recall period was collected in section 3. Food items were classified in 13 categories (cereals, lentils, fats, milk and milk products, sugar, meat, vegetables, fruits, beverages, alcoholic beverages, fried food, packaged food, and tea and coffee) and respondents were asked about expenditure in the last month and expected expenditure in the coming month. The classification follows the categorisation used by NSSO surveys, but we include disaggregated categories for beverages to measure spending on different temptation goods. This section deviated from standard food consumption expenditure; we collected information on expenditure incurred by the respondent and not for the household.

Section 4 focuses on the saving behaviour of the respondents. Detailed information regarding savings held at various institutions was collected. For different saving institutions such as bank, post office, and home, questions were asked about amount of savings at each of these institutions, and amount withdrawn and deposited in the past 30 days. Questions related to participation in informal saving associations were also included in this section.

In section 5, data for non-food household consumption expenditure was collected for 10 categories (tobacco consumption, lottery tickets or gambling, fares for transport, expense on fuel, religion, personal care, books and stationery, toys, repair and maintenance of the house, and any type of insurance). The questions and recall period were same as for section 3. Section 6 focused on expenditure behaviour. It included questions on health expenditure and unmet need for healthcare due to lack of financial resources, temptation spending behaviour and outstanding borrowings that the respondent owes to others. Section 7 tested the financial literacy of the respondent based on mathematical questions related to loan amounts and interest rates. Section 8 identifies if there are shops, kiosks, bars etc. around the respondent’s house or place of work that may trigger temptation spending.

Section 9 focused on eliciting attitudes towards gender roles and female empowerment. Five questions, presenting scenarios about what role males and females should play in a household, were presented to the respondent, for instance, husband should be more educated than their wife. The respondent answered on a 5-point likert scale indicating to what degree they agreed with the statement. Additionally, two questions related to freedom of movement were asked only to female respondents. The next section (section 10) relates to financial decision making within the household for female respondents. The female respondents were asked if they were involved in making financial decisions in the household, and the frequency at which financial issues create conflict with their partners and mothers-in-law.

### Survey instrument round 2

The survey instrument for round 2 was very similar to that from round 1 to ensure comparability in examining the effectiveness of the intervention. Except for section 7 and 8, where not much variation was expected in responses, all the sections from round 1 were retained in round 2. In section 4, additional questions were added regarding usage, usefulness, amount of savings, access and willingness to pay for the saving box and zip purse (only for treatment group) given to the respondents. Three additional questions on unexpected adverse financial shocks and ability to cope with them were added in section 6.

Section 7 and section 9 in this round correspond to section 9 and section 10 from round 1, respectively. An additional section (section 8) was added in round 2 to gain more information about how couples take decisions together. The respondent was presented with five hypothetical couples and information about how they make spending decisions. The respondent had to answer how similar they were to each of the hypothetical couple.

### Survey instrument round 3

Since data from round 3 was used to examine both research questions mentioned above, there were some sections that were common to survey instruments from round 1 and 2 while many new ones were added. Furthermore, many sections that were common were reduced to ensure that the questionnaire was not too long for a phone interview. Section 1 collected demographic information such as age, gender, education and employment. Additional questions about health condition of the respondent were added to under the percentage of respondents who suffer from co-morbidities such as diabetes, hypertension. In section 2, information about household demographic composition and living conditions such as access to sanitation and clean water was collected. Section 3 focused on COVID-19 related issues and included questions on COVID-19 knowledge and preventive behaviour, infections, economic and mental health difficulties faced by the respondent, and support received from private and public sources to deal with those difficulties. In section 4, information about exposure to media was collected to understand the role media can play in getting information about COVID-19. In section 5 respondent’s preferences related to time discounting, risk, honesty, and trust and trustworthiness were elicited through statement-based questions where respondents used a 10-point likert scale to show their degree of agreement with the statement. Information regarding decision making was collected in section 6 and corresponds to section 10 of previous rounds. Questions about attitudes towards domestic violence against the wife and child health care responsibilities were also included in this section. Respectively, section 7 corresponds to section 6, section 8 corresponds to asset part in section 2, and section 9 corresponds to section 3 of previous rounds and allows for comparison of savings, asset ownership and household food expenditure over time. Last section (section 10) of the survey instrument related to experience of domestic violence and was asked only to female respondents. This section was included in this survey round only.

### Survey instrument round 4

This survey instrument was quite similar to that in round 3. In section 1, questions about health conditions were dropped due to low variability in responses. In section 3, questions related to vaccination against COVID-19 were added. Section 7 (gender roles) and 11 (household non-food expenditure) from round 1 were added back, while section 10 from round 3 related to domestic violence was dropped.

## Data Records

The data for this study is available on the data depository platform, Göttingen Research Online, of University of Göttingen (10.25625/ODXY4G)^25^. There are a total of eight files, two files for each of the four rounds. Each file name includes the corresponding round number and year to clearly indicate which round they belong to. The questionnaire for each round is provided in English as PDF files, titled “questionnaire round [number] – [year]”. The data for each round is in MS Excel format, titled “raw data round [number] – [year]”. The data is in its raw format. Each of the four data files have three tabs: (1) dataset, (2) variable label, and (3) codebook. The first tab ‘dataset’ contains the raw data. The first column in tab ‘variable label’ provides the name of the variables and the second column presents the description of each variable. In the tab ‘codebook’, the response categories and their detailed descriptions are provided.

## Technical Validation

Pilot surveys were carried out to test the questionnaire before each round of data collection. The questionnaires were adjusted after analysing the data from the pilot surveys. Questionnaires were programmed using OpenDataKit, an open software for data collection. It allows inclusion of skip-patterns, reminders and consistency checks in the coded version of the questionnaire and improves data quality. Each completed questionnaire was checked and validated by field supervisors to maintain data quality. In case of implausible, odd or missing answers, field supervisors discussed with the enumerators and corrective steps such as contacting respondent again, were taken if necessary. Individuals were randomly assigned to treatment using the software STATA, and stratified by gender and baseline savings.

### Data collection and attrition

**Fig. 1:**
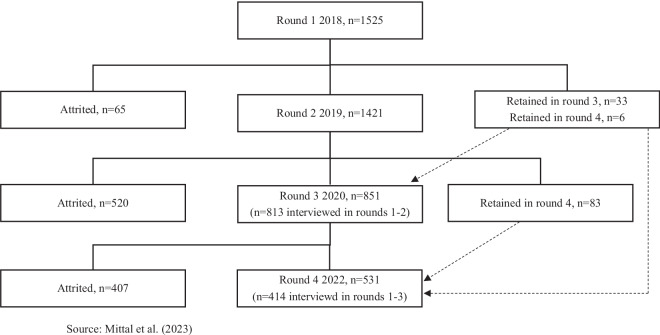
Sample size and attrition by survey round.

During the first two rounds, data was collected through household visits, while the last two rounds were conducted via phone interviews due to the COVID-19 pandemic. While the attrition was low between round 1 and round 2, it was significantly higher in last two rounds. Figure 1 presents the details of sample size and attrition for each round.

Data was collected for 1,525 slum dwellers in round 1 in December 2018-January 2019. The treatment was administered after the collection of baseline data. Respondents were randomly assigned to either treatment (771 respondents) or control group (754 respondents). Round 2 data was collected in August-October 2019 for 1421 respondents, implying an attrition of about 7 percent. Attrited respondents were more likely to be females, those who were employed or those who had higher incomes. In third round, conducted in October-December 2022, we could interview only 851 respondents from baseline (an attrition rate of 44 percent from round 1). The sample size further reduced to 531 respondents in round 4. The two primary reasons for attrition in last two rounds was inability to establish contact and refusal to participate in the study. In round 3 and 4, the respondents who participated in our phone survey were more educated, owned more assets and were more likely to be working as compared to average respondent from round 1. However, we have a balanced panel of 411 participants as not all individuals were interviewed in all four rounds. The balanced panel as compared to round 1 data, consists of more female, married, educated and working individuals (Please see refs. ^1,24^ for more details on characteristics of attrited respondents).

## Data Availability

The data for this study is available on the data depository platform, Göttingen Research Online, of University of Göttingen (10.25625/ODXY4G)^25^.

## Code availability

No custom code was used in generating the dataset.
